# An Atypical Initial Presentation of Systemic Lupus Erythematous With Bilateral Lower Extremity Edema and Unilateral Pleural Effusion

**DOI:** 10.7759/cureus.62091

**Published:** 2024-06-10

**Authors:** Su T Khine, Srujan Edupuganti, Mahpara Munir, Imad Modawi, Charles Swanson

**Affiliations:** 1 Internal Medicine, Hurley Medical Center, Michigan State University (MSU), Flint, USA; 2 Internal Medicine, Pediatrics, Hurley Medical Center, Michigan State University (MSU), Flint, USA; 3 Nephrology, Hurley Medical Center, Michigan State University (MSU), Flint, USA

**Keywords:** proteinuria, exudative pleural effusion, systemic lupus erythematosus, lupus, class v lupus nephritis

## Abstract

Systemic lupus erythematosus (SLE) is an autoimmune condition more commonly observed in women of childbearing age. The most commonly reported initial presentations were fatigue, arthritis, and skin manifestations. However, due to the involvement of a variety of organs, diagnosis remains a challenge for physicians. Our patient is a 48-year-old lady who presented with severe bilateral lower extremity edema with non-resolving right lower lobe pneumonia and ipsilateral exudative pleural effusion. Her leg swelling was not responding to diuretics, and her pneumonia was not improving following a course of antibiotics. This unusual presentation prompted an autoimmune workup, which later revealed a diagnosis of SLE with class 5 lupus nephritis. Pleuritis, exudative pleural effusion, and lupus nephritis have been associated with autoimmune disorders in the literature, but this is an uncommon initial presentation in SLE without other clinical manifestations. Our case report highlights the challenges in the diagnosis of an atypical case of SLE and the need to maintain high clinical suspicion for SLE, especially in female patients with multiorgan involvement.

## Introduction

Systemic lupus erythematosus (SLE) is a chronic inflammatory condition that can affect people of all ages and sexes but is especially common in women of childbearing age. According to the Lupus Foundation in the United States (US), there are estimated to be about 1.5 million cases of SLE. [1] SLE can affect multiple organs, mainly due to immune complex formation, deposition, and subsequent activation of the complement system. The most common initial presentations reported were fatigue (50%), arthritis or arthralgia (62-67%), and skin manifestations (73%), such as butterfly rash, photosensitivity, and alopecia. [2] The kidney is one of the organs that is commonly involved in SLE. Up to 38% of patients were noted to have lupus nephritis at the time of diagnosis. [3] The scope of involvement may range from urinary sediment to nephritic or nephrotic syndrome, which can lead to end-stage renal disease. In most cases, patients are diagnosed with SLE due to other clinical manifestations and later noted to have lupus nephritis. We are reporting a case of SLE with an atypical initial presentation of bilateral lower extremity edema, right-sided infiltrates, and pleural effusion without any skin or joint manifestations.

## Case presentation

A 48-year-old African American female with a history of essential hypertension and recently diagnosed atrial fibrillation presented to the emergency department (ED) with a complaint of bilateral lower extremity pedal edema and shortness of breath. She had two previous admissions at an outside facility before this presentation, the first one being three months prior, where she was treated for right lower lobe pneumonia, and the second one being a week prior, during which she presented with lower leg swelling and exertional dyspnea and was treated for heart failure. However, with diuresis, her symptoms did not improve, and she presented again to the ED. She denied coughing, fever, chest pain, or palpitations. She did report fatigue since she was first admitted for pneumonia. She had been compliant with antibiotics and diuretics.

**Figure 1:**
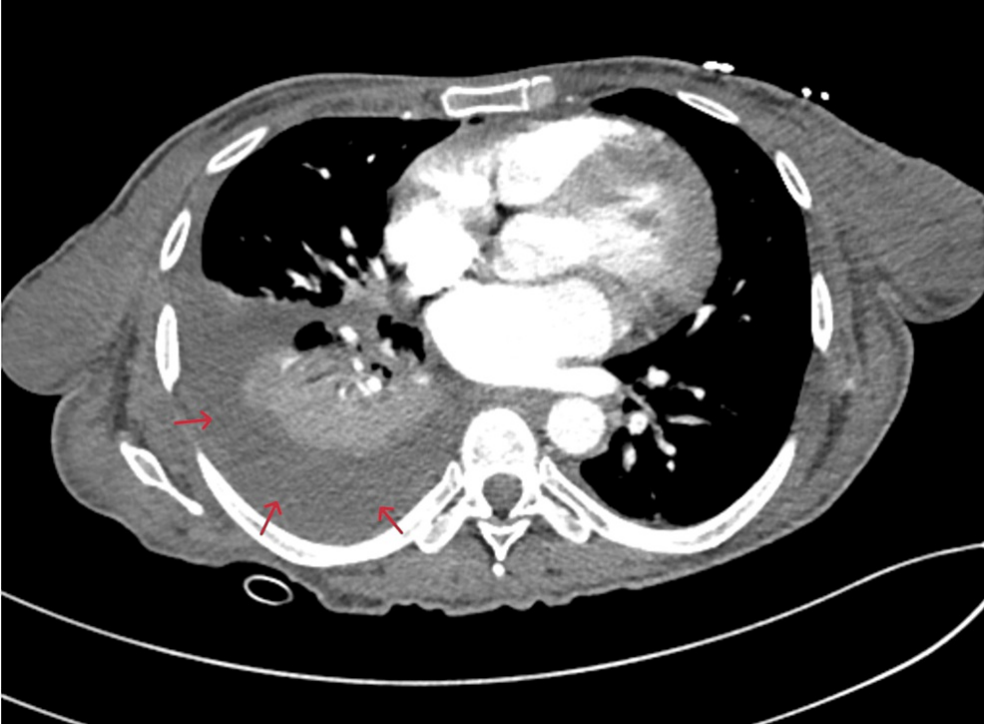
CT chest (axial view) shows dense consolidation in the right lower lobe and large right pleural effusion (red arrows).

On her initial presentation to the ED, she was hemodynamically stable and was breathing comfortably on room air. Blood work revealed chronic anemia, normal white counts, a mildly elevated B-type natriuretic peptide (BNP) of 204.5 pg/mL, and normal kidney function. The chest x-ray (CXR) showed right basilar pleural effusion with right lower lobe atelectasis and questionable infiltrates, raising concern for pneumonia. Hence, computerized tomography (CT) of the chest was done and was able to rule out pulmonary embolism, but was significant for a dense consolidation in the right lower lobe with a large right-sided pleural effusion that looked like a combination of pneumonia and atelectasis (Figure 1). A transthoracic echocardiogram revealed normal cardiac anatomy with an ejection fraction of 50-55% and normal diastolic function. She was later admitted to the medical floor. 

Both cardiology and pulmonology were consulted to assist in further management. The patient was initially started on intravenous (IV) furosemide twice daily for diuresis for two days, with little improvement in her leg swelling. Repeat chest radiography (CXR) on day three of the hospital stay was consistent with a worsening right basilar pleural effusion. Hence, right-sided ultrasound-guided thoracentesis was performed, and 600 mL of blood-tinged fluid was removed from the pleural space. She was started on empiric antibiotics with IV cefepime 2 g twice daily and IV metronidazole 500 mg thrice daily for concern for pneumonia. Pleural fluid analysis was consistent with exudative pleural effusion. Both aerobic and anaerobic cultures of the pleural fluid were negative, and cytological evaluation could not identify any malignant cells. As the clinical picture was not consistent with either heart failure or pneumonia, yet the patient continued to have severe bilateral lower extremity swelling, an extensive workup, including an autoimmune workup, was then done. 

The patient denied any rash, weight loss, bone or joint pain, or oral ulcers. Urinalysis (UA) showed 2+ protein with 5-10 RBCs. The serum albumin level was noted to be low at 2.7 g/dl. The urine protein/creatinine ratio was 0.8, with spot urine protein elevated at 145 mg/dl. Antinuclear antibody (ANA) came back positive with a titer of >1:1280 and a speckled pattern. Complement C3 and C4 were significantly low at 23 and 3 mg/dl, respectively. Results were negative for rheumatoid factor, anti-double-stranded DNA (DS-DNA) antibody, anti-histone antibody, and serum protein electrophoresis (SPEP). Nephrology was consulted for sub-nephrotic proteinuria with active urine sediments. Since the patient was on aspirin, the kidney biopsy was planned as an outpatient procedure. Thoracocentesis was repeated due to the re-accumulation of fluid in the right pleural space, and 400 ml of straw-colored fluid was drained. Following this, she was discharged home on torsemide 20 mg daily with a close follow-up with the nephrologist. 

**Figure 2:**
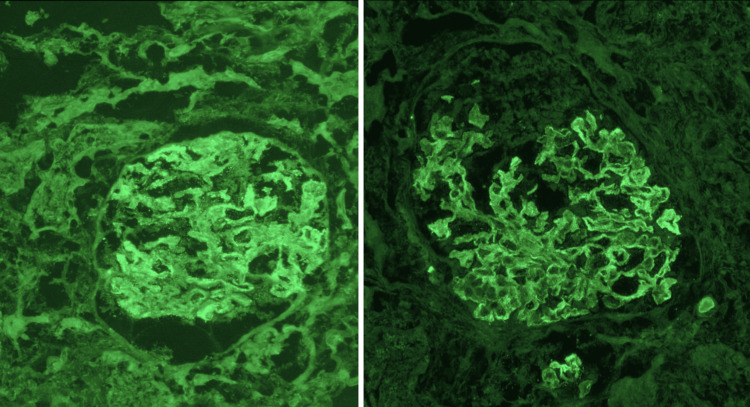
Immunofluorescence staining of a kidney biopsy was positive for C1q (left) and IgG (right).

**Figure 3:**
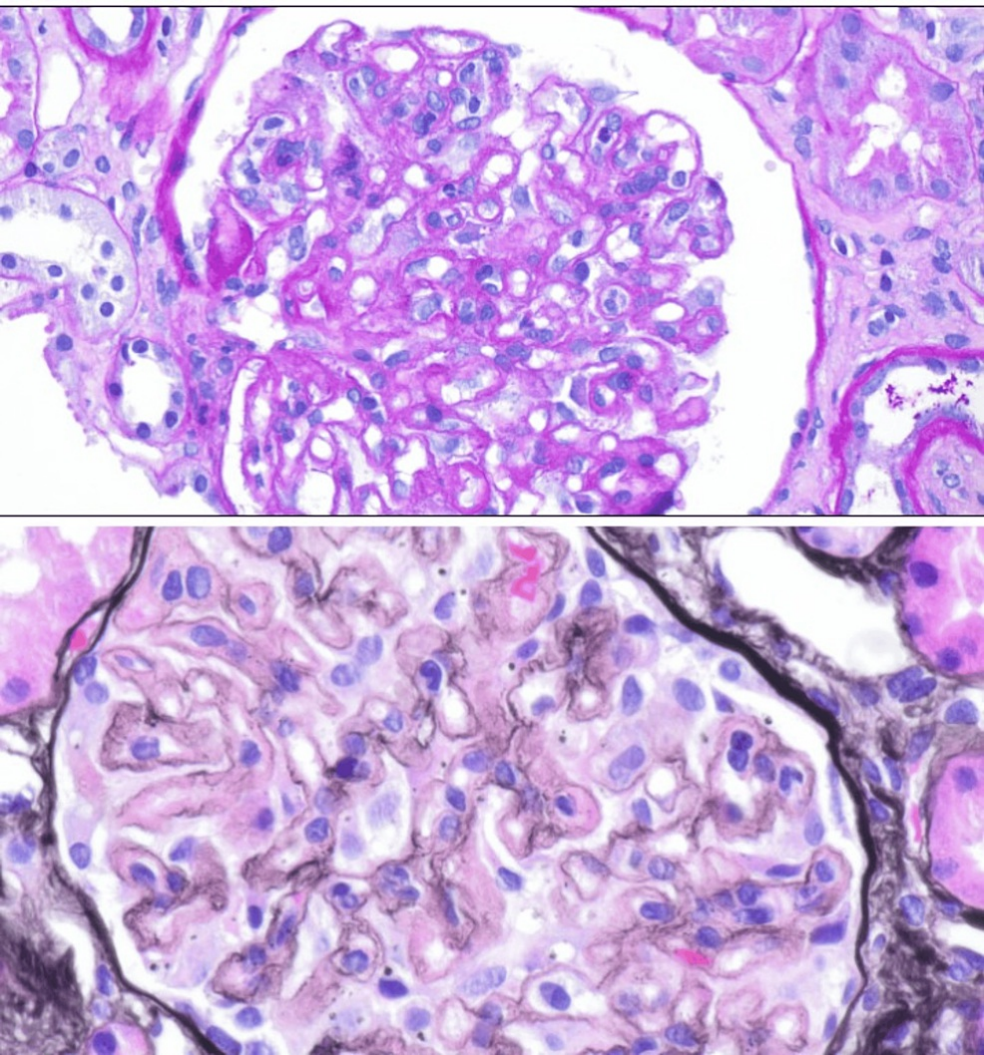
Light microscopy shows the endocapillary proliferation (top) and subendothelial hyaline deposits (bottom).

**Figure 4:**
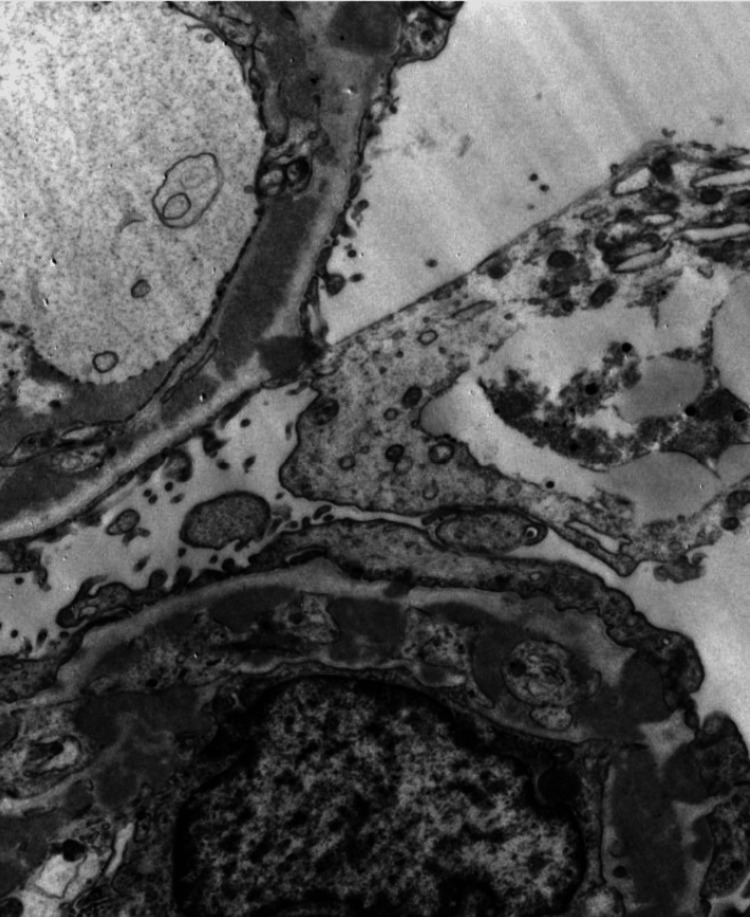
Electron microscopy of a kidney biopsy shows numerous subepithelial, intramembranous electron-dense deposits with numerous bulky mesangial deposits.

A kidney biopsy was done in an outpatient setting, and the histopathology was consistent with class 5 membranous nephropathy secondary to lupus nephritis (Figures 2-4). During outpatient follow-up with nephrology, the patient was subsequently started on prednisone 30 mg daily and mycophenolic acid 1.5 g twice a day, as well as angiotensin-receptor blockers and mineralocorticoid antagonists. 

## Discussion

SLE is an autoimmune disease with a female predominance that can present with a variety of clinical manifestations in any organ system. Given the broad and variable presentation of the disease, diagnosing it remains a challenge for physicians. The American College of Rheumatology (ACR) [4] and Systemic Lupus International Collaborating Clinic (SLICC) classification criteria [5] can also be used as a reference in the diagnosis of SLE, yet the presentations can be very diverse and the diagnosis of SLE requires high clinical suspicion and judgment by physicians. 

Even though serositis and exudative pleural effusion are usually associated with autoimmune disorders such as lupus and rheumatoid disease, an isolated finding poses difficulty in considering autoimmune disease as a differential. In our case, the patient was treated for pneumonia prior to presentation and was now noted to have right lower lobe consolidation on initial imaging with a newly developed pleural effusion on the same side, which led to the misdiagnosis of recurrent pneumonia. In addition, negative pleural fluid culture can also be seen in parapneumonic effusion. Although infections have been associated with SLE, they are usually found in the later stages of the disease due to the use of immunosuppressive therapy. [2] Here, there was no recurrent pneumonia but instead atelectasis from pleural effusion. Acute lupus pneumonitis is another rare complication of lupus that can present similarly to pneumonia. However, the condition typically presents with bilateral lung infiltrates, which was not the case in our patient. [6]. Another unusual presentation in our patient was that she had concurrent severe bilateral lower extremity edema and mild proteinuria, which cannot be explained by pneumonia, leading to the consideration of autoimmune disease as a differential. 

Lupus nephritis is a very commonly associated consequence of SLE. Again, diagnosing lupus nephritis can be difficult because of the wide range of clinical manifestations. Typically, patients are asymptomatic, and lupus nephritis is discovered due to abnormal urinary sediments or renal function after an SLE diagnosis based on other clinical symptoms. [7] In our case, the patient did not manifest skin, mucosal, or joint problems, and her initial chief complaint was the worsening lower extremity edema, which is not responding to diuretics. However, her urinalysis showed proteinuria and hematuria along with a low albumin level, and her echocardiogram with a mildly reduced ejection fraction also did not correspond with the degree of her clinical symptoms. Therefore, further evaluation of the kidney disorder was done in her case, and she was subsequently diagnosed with lupus nephritis. This case report highlights that it is important for clinicians to consider renal pathology in differentials, especially in the setting of severe bilateral lower extremity edema and hypoalbuminemia. 

Knowledge of the ACR guidelines for SLE diagnosis is also helpful in triggering the necessary workup. SLE can be very challenging to diagnose, which requires physicians to maintain high clinical suspicions, especially when the patients are presenting with multiorgan system involvement. It is additionally important to diagnose SLE early, as the prognosis can be worse in Black, Asian, and Hispanic individuals. In addition, the presence of lupus nephritis can negatively affect the prognosis, morbidity, and mortality, again pointing to the need for early diagnosis [3,8].

## Conclusions

Exudative pleural effusion in appropriate clinical settings should prompt clinicians to consider autoimmune disorders as differentials. Severe bilateral lower extremity edema, proteinuria, and hypoalbuminemia should raise the clinical suspicion of nephrotic syndrome. Lupus nephritis is a commonly found association that may or may not be present at initial presentation but can determine the prognosis of SLE. In SLE cases, clinicians should closely monitor urinary sediments.

## References

[1] (2024). How Many People Have Lupus in the United States? | Lupus Foundation of America.. https://www.lupus.org/resources/how-many-people-have-lupus-in-the-united-states.

[2] (2024). Symptoms of SLE. https://www.uptodate.com/contents/image.

[3] Hanly JG, O'Keeffe AG, Su L (2016). The frequency and outcome of lupus nephritis: results from an international inception cohort study. Rheumatology (Oxford).

[4] Aringer M, Costenbader K, Daikh D (2019). 2019 European League Against Rheumatism/American College of Rheumatology classification criteria for systemic lupus erythematosus. Arthritis Rheumatol.

[5] Petri M, Orbai AM, Alarcón GS (2012). Derivation and validation of the Systemic Lupus International Collaborating Clinics classification criteria for systemic lupus erythematosus. Arthritis Rheum.

[6] Cantero C, Vongthilath R, Plojoux J (2020). Acute lupus pneumonitis as the initial presentation of systemic lupus erythematosus. BMJ Case Rep.

[7] Musa R, Brent LH, Qurie A (2023). Lupus Nephritis. StatPearls [Internet].

[8] Almaani S, Meara A, Rovin BH (2017). Update on lupus nephritis. Clin J Am Soc Nephrol.

